# The design and manufacture of massively scalable inertial focusing prototype microfluidic devices

**DOI:** 10.1371/journal.pone.0324434

**Published:** 2025-12-05

**Authors:** Thomas Carvell, Paul Burgoyne, Alasdair R. Fraser, Helen Bridle

**Affiliations:** 1 Institute of Biological Chemistry, Biophysics and Bioengineering, School of Engineering and Physical Sciences, Heriot-Watt University, Edinburgh, United Kingdom; 2 Tissues, Cells and Advanced Therapeutics, Jack Copland Centre, Scottish National Blood Transfusion Service, Edinburgh, United Kingdom; Università Campus Bio-Medico di Roma, ITALY

## Abstract

Microfluidics is a rapidly expanding field and microfluidic devices have been used in a variety of biomedical applications such as cell sorting, disease diagnostics and various lab-on-a-chip systems. There is great demand for manufacturing techniques capable of fabricating ever more intricate microstructures for increasingly complex applications whilst remaining cost-effective for use in biomedical research. Conventional manufacturing techniques can be used to fabricate many complex microchannel architectures but are often expensive, low throughput, have poor microfeature resolution or are unsuitable to be used at scale. To address this issue, we describe a manufacturing technique that employs stereolithography 3D printing to produce a base substrate which can be sealed with a laser patterned adhesive layer cover and stacked in a compact configuration. The technique provides for the design and manufacturing of a device that can be massively parallelized at relatively low cost and with a small laboratory footprint. This paper aims to fully explain the design and manufacturing process undertaken to allow the use of this technique in future research.

## Introduction

Microfluidics has shown enormous potential for a wide variety of applications and is used in a diverse range of industries today. Inertial focusing (IF) microfluidic devices can manipulate cells within microfluidic systems and have been used to separate target cells, reduce buffer volume and have also been integrated with sensors for diagnostic purposes [1–3].

**Fig 1:**
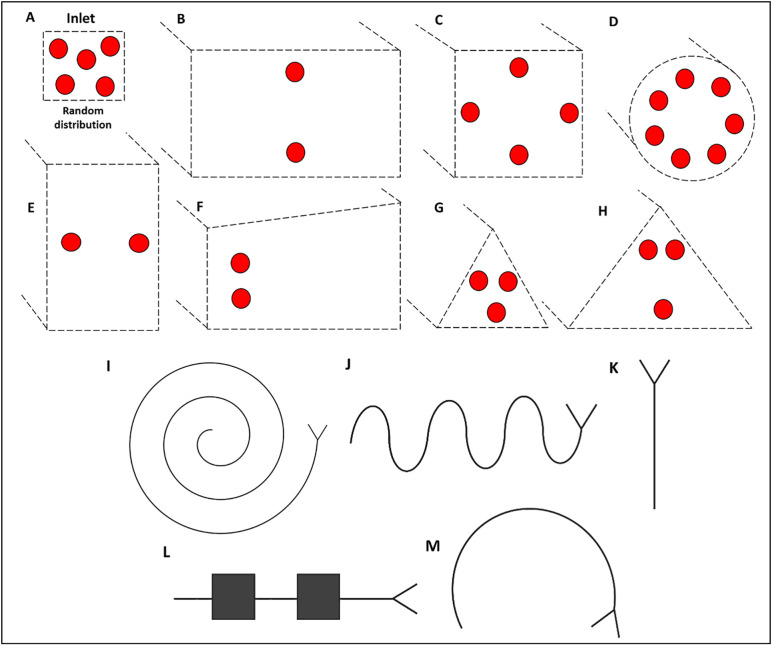
Schematic illustrative particle focal positions and microchannel design flexibility. **A)** Cells are distributed randomly at the cross-section of the inlet microchannel. Illustrative focal positions* of cells in IF microchannels with the following cross sections: **B)** Rectangular, **C)** square, **D)** circular, **E)** rectangular, **F)** trapezoidal, **G)** narrow-bottomed triangle, **H)** wide-bottomed triangle. Top-down view of schematics of IF microchannels with different designs and a Y-junction outlet: **I)** spiral, **J)** serpentine, **K)** straight, **L)** expansion-contraction arrays **M)** curved. * Based on specific fluid flow characteristics.

Due to their compact size, design flexibility, and reproducibility, microfluidic devices are attractive alternatives to conventional cell processing equipment [4,5]. Illustrative focal positions in microchannels with different geometry are shown in Fig 1.

IF devices process cells without centrifugation, labelling, or filtration, and utilize only the hydrodynamic forces generated by the geometry of microchannels for their mechanism of action [5]. Details of IF and other forces that are generated in microfluidic devices are well reviewed [3,5–9].

Experimentation with novel microchannel structures is therefore highly desirable but current techniques to manufacture these are either too expensive, have poor resolution or are incompatible with high-throughput processing [10,11]. Predicting fluid flow within microchannels is highly complex [12] but experimentalists can use trial and error to optimize the processing of various materials. The adoption of inexpensive fabrication techniques that can be used to produce complex microchannel geometries would therefore greatly support the growing field of microfluidics.

The microchannel architecture is of paramount importance and there are many significant parameters involved with IF [6,8]. Processing with IF devices is complex due to the interplay between various forces [13]. Many factors require consideration, but for simplicity, a select few have been considered for this protocol. First, IF only occurs within a laminar flow regime with a channel Reynold’s number (Re_c_) that fulfils 1 > Re < 2000 [14], as per the following:


$$Re_{c}=\frac{\rho U_{Max}D_{h}}{\mu }$$

where ρ and µ are the density and viscosity of fluid, U_Max_ is the maximum channel velocity and D_h_ is the hydraulic diameter of the channel.

Second, the channel cross-section is a key determinant of cell focusing position and for successful IF, the following criterion [15,16] should be met:


$$\frac{a_{p}}{\text{0}.\text{5}}\leq h\leq \frac{a_{p}}{\text{0}.\text{0}\text{7}}$$

where α_p_ is the average cell diameter and h is the height of the microchannel. Changing the cross-section of the microchannel can dramatically alter the profile of the flow and therefore microchannels with complex geometries such as a trapezoidal cross-sections have been investigated for different applications [17].

Third, the channel width can be determined based on the channel aspect ratio, which greatly impacts focusing [6], and as this would be application-dependent, for this protocol, an arbitrary aspect ratio of 6:1 (W/H) was selected.

Finally, the channel length required for particle focusing can be estimated in a straight channel by determining [13]:


$$L_{\text{f}}=\frac{\pi \mu h^{\text{2}}}{\rho {⋃}_{Max}a^{\text{2}}f_{L}}$$

where L_f_ is the lateral equilibrium position. The addition of curvature affects the required length of channel and can be estimated through the multiplication of the value for the straight channel length equation above where ƒ = 0.2–1 [13]. The addition of curvature to the microchannel induces secondary flows that generate Dean flow and cause a recirculation of the fluid orthogonal to the direction of flow and can assist with cell separation [18]. However, where the ratio of inertial lift to Dean drag forces is much higher than 1, the secondary Dean flow which can be used to enhance cell separation whilst reducing channel length, becomes negligible [13].

**Fig 2:**
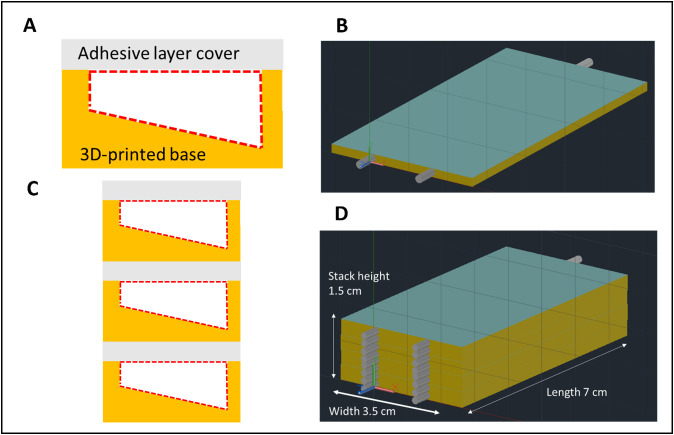
Designs for stacking mechanism of microfluidic device. **A)** schematic showing cross-section of a single device and **B)** CAD drawing of prototype single device. **C)** Schematic diagram showing the cross-section stacking and **D)** CAD drawing showing the potential for a massively parallelized prototype technique.

The modelling and prediction of just a few factors of IF processing is challenging and experimental work is essential. To address the difficulties of manufacturing complex architectures, we describe a protocol for the design and fabrication of microfluidic devices with a specific focus on IF microfluidics. The critical stages of the manufacturing process utilize both laser ablation to pattern adhesive tape, and stereolithography 3D printing for constructing the microchannel base layer. When sealed together, this process allows for the manufacture of devices that can be easily stacked into a compact size (Fig 2), allowing for mass parallelization not possible with many other device designs. Whilst microfluidic devices fabricated through the 3D-printing of bases, sealed with an adhesive tape cover have been previously reported [19], this design differs because the outlets and tubing are orientated parallel to the flow channel and therefore massively minimises the laboratory footprint when devices are stacked without complicated inlet and outlet tubing attachments. Each device is 3.05 mm in thickness (4.05 mm if seeking a more robust device with an acrylic cover) and material costs of ~£1.20 (~$1.50 as of August 2024), making this design ideal for resource and space-limited research environments. Other low-cost ‘do-it-yourself’ style microfluidic devices have been previously reported, including those used for paper-based urine analysis [20] and hydrophobic barrier or those employing electrodes [21].

This manufacturing process can be used for many applications within microfluidics, but this protocol will provide a specific example through the design and manufacturing of an IF device but will be compared to two other devices to illustrate design flexibility, including a more complex device containing microchannel with a trapezoidal cross-section. Specific manufacturing parameters are detailed and selected results using this technique are reported and discussed.

## Materials and methods

The protocol described in this peer-reviewed article is published on protocols.io, dx.doi.org/10.17504/protocols.io.n2bvjnrmpgk5/v1 and is included for printing as supporting information file 1 with this article.

It should be noted that the protocol describes a method to fabricate microfluidic devices with a wide range of microchannel geometries and exemplar devices are described. The characterisation steps (such as flow rate and particle concentration) also are highly dependent on both the device and application. The use of a haemocytometer was employed to demonstrate quantification of particles in a resource-limited environment, but automated systems such as flow cytometry can also be used. The separation efficiency was calculated by dividing the number of particles from individual outlet counts by the total number of particles in both outlet counts.

## Expected results

**Fig 3:**
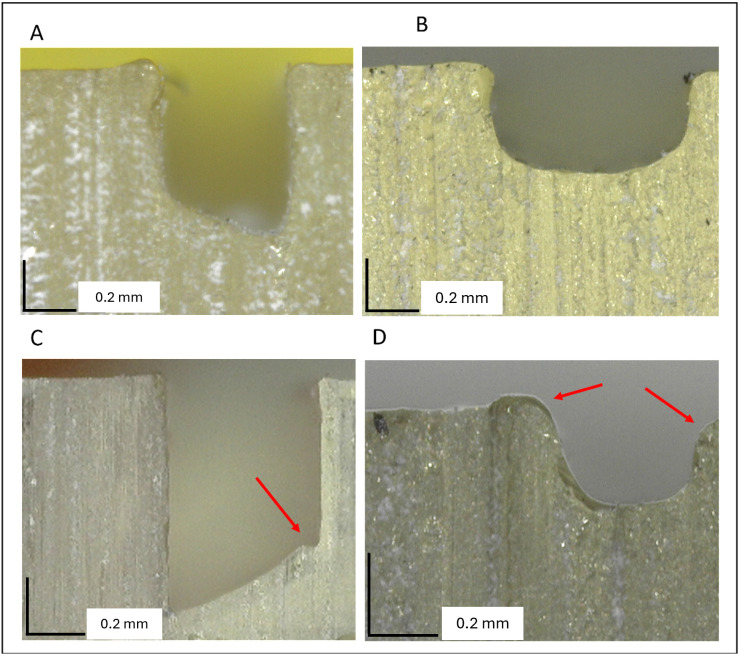
Microscopic images of cross-sections of 3D-printed base layers. The addition of an adhesive cover results in A) trapezoidal and B) rectangular straight channels. Imperfections (red arrows) in a C) trapezoidal cross-section and a D) rectangular straight channel.

When using this protocol, the user should be aware that the manufacturing of microfluidic devices with complex microchannel geometries can be a challenging process. The protocol describes the fabrication of a straight microchannel with a rectangular cross-section, but the protocol is compatible with the design of a wide range of complex microchannel cross-sections. Further designs have been uploaded to the repository. To assist the user of this protocol, examples of microfluidic devices with the correct and incorrect microchannel geometries have been imaged (Fig 3).

Microfluidic devices with two different cross-sections were manufactured using the protocol and trapezoidal and rectangular cross-sections were imaged and both positive (Figs 3A + B) and negative results are shown (Figs 3C + D). The imperfections in the microchannel shown in Figs 3C + D will cause disturbances to the flow that could impact device function and should be discarded.

**Fig 4:**
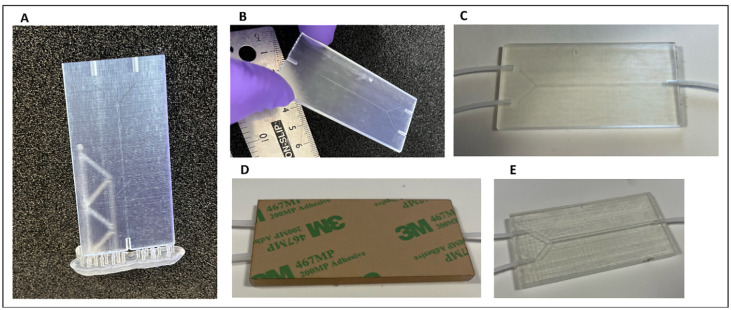
Images of device assembly process. **A)** The finished 3D-printed base with build supports. **B)** The supports are removed and **C)** PFTE tubing inserted in the ports and adhesive used to secure the tubing in place. **D)** The adhesive tape covers the 3D-printed base and **E)** the backing removed.

Imaging showing the assembly of a device using this protocol is shown in Fig 4.

**Fig 5:**
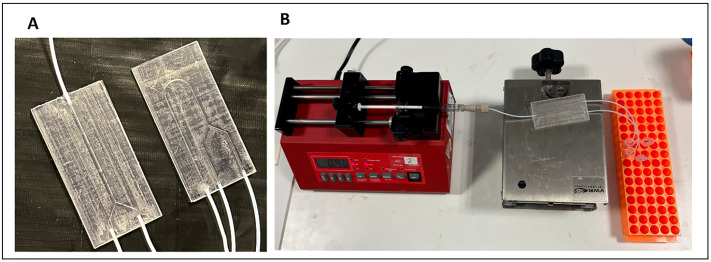
A) Fully assembled microfluidic devices with straight channel (left) and curved channel (right). B) The processing system experimental set up showing the syringe pump injecting particle suspensions into a device and flow collected at the outlets in microcentrifuge tubes.

Once assembled, three exemplar devices were attached to a syringe pump and particle suspensions were injected at the inlets (Fig 5).

**Fig 6:**
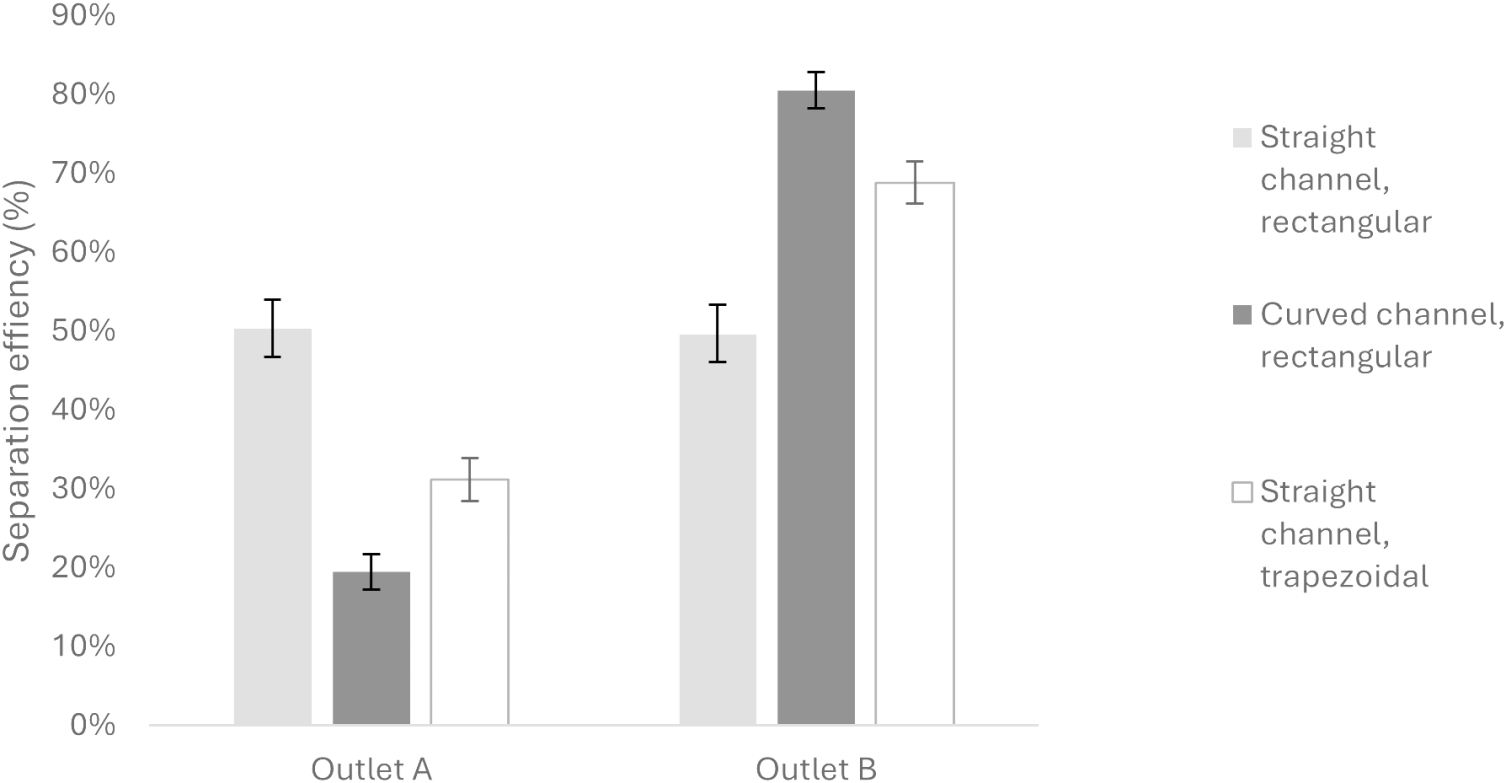
Chart showing representative particle separation to outlet A or outlet B in devices with straight channel (with rectangular cross-section), curved (with rectangular cross-section) and straight channel (with trapezoidal cross-section) at a flow rate of 0.9 mL/min.

The flow was collected at the outlets and the outlet samples were quantified using a hemacytometer and the data are shown in Fig 6. At certain flow conditions, particles undergo inertial focusing and reach equilibrium positions at centre of the cross-section of a straight microchannel with a rectangular cross-section (Fig 1B). The addition of curvature (to a microchannel with rectangular cross-section) generates secondary forces known as Dean flow that can result in off-centre equilibrium positions and therefore particles can be collected at one outlet. In straight channel microchannels with trapezoidal cross-sections, Dean flow also occurs and off-centre focusing positions can also be utilised to collect particles at specific outlets. With these exemplar devices, the particles are randomly separated at the outlets in the straight channel but are predominantly separated to outlet B in the curved device. It should be noted that the separation of particles is highly dependent on numerous factors and these data, whilst generated using manufactured devices, should be regarded as illustrative only. This protocol is described primarily to aid researchers in the design of a highly customisable microfluidic device capable of massive parallelisation.

In summary, this manufacturing process requires further optimization to enable the fabrication of ever more complex microstructures, but this protocol is an excellent candidate for wide-scale adoption because the technologies are very accessible, it has design flexibility, huge scalability, very low consumable costs and exceptional performance.

### Associated content

The step-by-step laboratory protocol is available at dx.doi.org/10.17504/protocols.io.n2bvjnrmpgk5/v1.

Data and design files are available at https://figshare.com/projects/The_design_and_manufacture_of_massively_scalable_inertial_focusing_prototype_microfluidic_devices/219574.

## Supporting information

S1 File Step-by-step protocol, also available on protocols.io. (PDF)

## References

[1] Niculescu A-G, Chircov C, Bîrcă AC, Grumezescu AM. Fabrication and applications of microfluidic devices: a review. Int J Mol Sci. 2021;22(4):2011. doi: 10.3390/ijms22042011 33670545 PMC7921936

[2] Shiriny A, Bayareh M. Inertial focusing of CTCs in a novel spiral microchannel. Chemical Eng Sci. 2021;229:116102. doi: 10.1016/j.ces.2020.116102

[3] Shields CW 4th, Reyes CD, López GP. Microfluidic cell sorting: a review of the advances in the separation of cells from debulking to rare cell isolation. Lab Chip. 2015;15(5):1230–49. doi: 10.1039/c4lc01246a 25598308 PMC4331226

[4] Lu M, Lezzar DL, Vörös E, Shevkoplyas SS. Traditional and emerging technologies for washing and volume reducing blood products. J Blood Med. 2019;10:37–46. doi: 10.2147/JBM.S166316 30655711 PMC6322496

[5] Huang D, Man J, Jiang D, Zhao J, Xiang N. Inertial microfluidics: recent advances. Electrophoresis. 2020;41(24):2166–87. doi: 10.1002/elps.202000134 33027533

[6] Martel JM, Toner M. Inertial focusing in microfluidics. Annu Rev Biomed Eng. 2014;16(1):371–96. 24905880 10.1146/annurev-bioeng-121813-120704 PMC4467210

[7] Cruz F, Zadeh SH, Wu Z, Hjort K. Inertial focusing of microparticles and its limitations. J Phys: Conf Ser. 2016;757:012028. doi: 10.1088/1742-6596/757/1/012028

[8] Zhang J, Yan S, Yuan D, Alici G, Nguyen N-T, Ebrahimi Warkiani M, et al. Fundamentals and applications of inertial microfluidics: a review. Lab Chip. 2016;16(1):10–34. doi: 10.1039/c5lc01159k 26584257

[9] Convery N, Gadegaard N. 30 years of microfluidics. Micro and Nano Engineering. 2019;2:76–91. doi: 10.1016/j.mne.2019.01.003

[10] Venkatesan S, Jerald J, Asokan P, Prabakaran R. A comprehensive review on microfluidics technology and its applications. Singapore: Springer Singapore; 2020.

[11] Scott SM, Ali Z. Fabrication methods for microfluidic devices: an overview. Micromachines (Basel). 2021;12(3):319. doi: 10.3390/mi12030319 33803689 PMC8002879

[12] Abidi A, Ahmadi A, Enayati M, Sajadi SM, Yarmand H, Ahmed A, et al. A review of the methods of modeling multi-phase flows within different microchannels shapes and their applications. Micromachines (Basel). 2021;12(9):1113. doi: 10.3390/mi12091113 34577756 PMC8465032

[13] Amini H, Lee W, Di Carlo D. Inertial microfluidic physics. Lab Chip. 2014;14(15):2739–61. doi: 10.1039/c4lc00128a 24914632

[14] Duncombe TA, Tentori AM, Herr AE. Microfluidics: reframing biological enquiry. Nat Rev Mol Cell Biol. 2015;16(9):554–67. doi: 10.1038/nrm4041 26296163 PMC6240156

[15] Bhagat AAS, Kuntaegowdanahalli SS, Papautsky I. Inertial microfluidics for continuous particle filtration and extraction. Microfluid Nanofluid. 2008;7(2):217–26. doi: 10.1007/s10404-008-0377-2

[16] Xiang N, Shi Z, Tang W, Huang D, Zhang X, Ni Z. Improved understanding of particle migration modes in spiral inertial microfluidic devices. RSC Adv. 2015;5(94):77264–73. doi: 10.1039/c5ra13292d

[17] Moloudi R, Oh S, Yang C, Ebrahimi Warkiani M, Naing MW. Inertial particle focusing dynamics in a trapezoidal straight microchannel: application to particle filtration. Microfluid Nanofluid. 2018;22(3). doi: 10.1007/s10404-018-2045-5

[18] Ramachandraiah H, Ardabili S, Faridi AM, Gantelius J, Kowalewski JM, Mårtensson G, et al. Dean flow-coupled inertial focusing in curved channels. Biomicrofluidics. 2014;8(3):034117. doi: 10.1063/1.4884306 25379077 PMC4162445

[19] Qiu J, Li J, Guo Z, Zhang Y, Nie B, Qi G, et al. 3D Printing of individualized microfluidic chips with DLP-based printer. Materials (Basel). 2023;16(21):6984. doi: 10.3390/ma16216984 37959581 PMC10650121

[20] Tai WC, Chang YC, Chou D, Fu LM. Lab-on-paper devices for diagnosis of human diseases using urine samples—A review. Biosensors. 2021;11(8):260. 34436062 10.3390/bios11080260 PMC8393526

[21] Singhal HR, Prabhu A, Giri Nandagopal MS, Dheivasigamani T, Mani NK. One-dollar microfluidic paper-based analytical devices: do-it-yourself approaches. Microchemical Journal. 2021;165:106126. doi: 10.1016/j.microc.2021.106126

