# Supplementary material for: The design and manufacture of massively scalable inertial focusing prototype microfluidic devices
Source: PLoS One. 2025 Dec 5;20(12):e0324434. doi: 10.1371/journal.pone.0324434 (PMC12680145; doi:10.1371/journal.pone.0324434)
Supplement: S1 File — (PDF) [file pone.0324434.s001.pdf]

Apr 06, 2024 Version 1

The design and manufacture of massively scalable inertial focusing prototype microfluidic devices V.1

DOI
dx.doi.org/10.17504/protocols.io.n2bvjnrmpgk5/v1

Thomas Carvell[1]

[1]Heriot-Watt University

Thomas Carvell
Heriot-Watt University

Create & collaborate more with a free account

Edit and publish protocols, collaborate in communities, share insights through comments, and track progress with run records.

Create free account

OPEN ACCESS

DOI: **https://dx.doi.org/10.17504/protocols.io.n2bvjnrmpgk5/v1**

Protocol Citation: Thomas Carvell 2024. The design and manufacture of massively scalable inertial focusing prototype microfluidic devices. **protocols.io** **https://dx.doi.org/10.17504/protocols.io.n2bvjnrmpgk5/v1**

License: This is an open access protocol distributed under the terms of the **Creative Commons Attribution License**, which permits unrestricted use, distribution, and reproduction in any medium, provided the original author and source are credited

Protocol status: Working
We use this protocol and it's working

Created: April 04, 2024

Last Modified: April 06, 2024

Protocol Integer ID: 97787

Keywords: 3D printing, microfluidic device manufacturing, laser ablation, high throughput, microfluidic device, microfluidic devices microfluidic, prototype microfluidic devices microfluidic, microfluidic, stereolithography 3d printing, many complex microchannel architecture, manufacturing technique, great demand for manufacturing technique, poor microfeature resolution, intricate microstructure, manufacturing process, conventional manufacturing technique, variety of biomedical application, biomedical application, manufacturing, small laboratory footprint, chip system, patterned adhesive layer cover, focusing prototype, substrate, base substrate, biomedical research

Funders Acknowledgements:
Medical Research Scotland
Grant ID: 50167-2019

Abstract

Microfluidics is a rapidly expanding field and microfluidic devices have been used in a variety of biomedical applications such as cell sorting, disease diagnostics and various lab-on-a-chip systems. There is great demand for manufacturing techniques capable of fabricating ever more intricate microstructures for increasingly complex applications whilst remaining cost-effective for use in biomedical research. Conventional manufacturing techniques can be used to fabricate many complex microchannel architectures but are often expensive, low throughput, have poor microfeature resolution or are unsuitable to be used at scale. To address this issue, we describe a manufacturing technique that employs stereolithography 3D printing to produce a base substrate which can be sealed with a laser patterned adhesive layer cover and stacked in a compact configuration. The technique provides for the design and manufacturing of a device that can be
massively parallelized at relatively low cost and with a small laboratory footprint. This paper aims to fully explain the design and manufacturing process undertaken to allow the use of this technique in future research.

Guidelines

- Computer-aided design (CAD) software should be used to design both the 3D-printed and laser patterned parts.
- When using the stereolithography 3D-printer, follow the manufacturer's instructions and any local safety regulations.
- When using a CO_2 laser cutter, follow the manufacturer's instructions and any local safety regulations.

Materials

	A	B	C	D
	**Name of Material/ Equipment**	**Company**	**Catalog Number**	**Comments/Description**
	29.32 µm particles	Microparticles GmbH	PS-R-30.0	N/A
	1000 µL pipette tips	FisherSci	11710645	N/A
	50 µm thick double-sided adhesive tape	3M	467MP	N/A
	50 mL conical tubes	FisherSci	10788561	N/A
	AutoCAD 2019	AutoDesk	N/A	Software
	Cell Strainer	FisherSci	10737821	N/A
	Clear Resin	FormLabs	RS-F2-GPCL-04	N/A
	dH20	FisherSci	11508886	N/A
	Eppendorfs	FisherSci	10154671	N/A
	Form2 3D printer	FormLabs	N/A	Form3 is a suitable alternative but different accessories are required and optimisation steps will likely be required
	Form 2 Biomed Clear resin 1 L	FormLabs	RS-F2-BMCL-01	N/A
	Form 2 Resin Tank	FormLabs	RT-F2-01	Wiper included.
	Isopropranol	Sigma Aldrich	PX1830	N/A
	mini 18 Laser System	Epilog	8000	Helix 24 is a suitable alternative
	PFTE tubing	PFTE Tube shop	PTFE tube 0.8 x 1.2	Catalog number is SKU.

A	B	C	D
Phosphate Buffered Saline	FisherSci	15313581	N/A
Pipette	FisherSci	11887351	N/A
Pipette controller	FisherSci	15850053	N/A
Rocking platform	FisherSci	15995911	N/A
Sample bag	FisherSci	13554530	N/A
Stainless Steel Build Platform	FormLabs	BP-CBSS-02	N/A
Stripette	FisherSci	10041591	N/A
Syringes	FisherSci	12921031	N/A
Haemocytometer	FisherSci	11704939	N/A
Syringe pump	World precision instruments	AL-1010	N/A

Table of materials.

Troubleshooting

Safety warnings

Always ensure only properly trained individuals operate laser systems and equipment as per local safety regulations.

Ethics statement

Not applicable.

Before start

Read the entire protocol before starting. Note: section 1 describes the design process for a specific, but exemplar, microfluidic device. Steps 1.6 to 1.33 can be modified as appropriate for other applications.

The characterisation of the device was performed to provide representative results only and does not need to be followed. This will highly depend on the final application of the device that can be manufactured using this protocol.

Design of exemplar parts

1 Open computer-aided design software and ensure 3D-modelling format is selected and units are set to millimetres.

1.1 Select the rectangle tool and enter x-y coordinates for origin (0, 0). The width is 35 and the height is 70. This generates the 'device rectangle'.

1.2 Save the device rectangle as 'adhesive tape cover' using the .dxf file type. Note: no further modifications to this file are needed.

1.3 Save the device rectangle as '3D printed base'.

1.4 Open the '3D printed base' CAD file.

1.5 Select the line tool and draw a line (line A) from the midpoint of the top side of the rectangle at 90° and 15 in length.

1.6 Repeat step 1.3 for the bottom side at 90° and 15 in length to generate line B.

1.7 Use the line tool and draw a line (line C) of 0.25 in length and at an angle of 180° relative to the central endpoint of line A.

1.8 Use the line tool and draw a line (line D) of 0.25 in length and at an angle of 0° relative to the central endpoint of line B.

1.9 Use the rectangular tool to draw a rectangle from the non-centred endpoints of line C and line D. Note: the rectangle (microchannel rectangle) should be 40 in length and 0.5 in width.

1.10 Delete lines A, B, C and D.

1.11 Using the line tool, draw a line (135° angle, 10 in length) from the midpoint of the top side of the microchannel rectangle.

1.12 Draw another line (45° angle, 10 length) from the same midpoint as in step 1.12.

1.13 Draw two lines from the bottom of the microchannel rectangle and extend these to the device rectangle side (both lines, 90°, 15 in length).

1.14 Use the offset tool to generate parallel lines (0.25 distance away) to the lines drawn in steps 1.12 and 1.13. There will be some lines that overlap with the microchannel rectangle, but a Y-shaped channel will be displayed.

1.15 Use the trim tool to remove any lines inside the Y-shaped microchannel and remove all parts of the microchannel rectangle drawing that protrude past the offset lines. This should generate a central channel with an inlet channel and 2 smaller outlet channels at a Y-shaped angle.

1.16 Use the line tool and draw a line (90° angle, 10 in length) from each of the outlet channel lines. These will extend past the edge of the device rectangle.

1.17 Use the trim tool to remove all extended lines drawn in step 1.16.

1.18 Connect the end of all channels. These should be 0.1768 in length for the smaller outlet channels and 0.5 in length for the inlet channel.

1.19 Select all lines except for the device rectangle and use the join command. This should join 14 lines into one polyline.

1.20 Select the device rectangle and extrude by 3, below the plane of the Y-shaped channel. This forms the device cuboid.

1.21 Select the Y-shaped polyline and extrude by 0.15 below the plane of the polyline.

1.22 Use the Face tool and select the cross-section of the microchannel.

1.23 Use the rectangle tool to draw a rectangle (with the same dimensions as the relevant channel cross-section) on each face of the end of the inlet and outlet channels.

1.24 Draw a line below the channel plane (0.9 length, angle 90°) from each of the center-points of the 3 rectangles drawn in step 1.24.

1.25 Delete each of the rectangles drawn in step 1.24.

1.26 At the bottom endpoint of each of the three lines (drawn in step 1.25) draw a circle with 0.9 radius.

1.27 Delete the lines drawn in step 1.25.

1.28 Select each of the circles from step 1.24 and use the extrude tool to extrude the circle by 5 towards the centre of the device to form cylinders.

1.29 Select the subtract tool and left click the device cuboid.

1.30 Right click anywhere outside of the design.

1.31 Left click the Y-shaped channel and the cylinders.

1.32 Right click anywhere outside of the design.

1.33 To confirm this has successfully removed the cylinders and channel from the device cuboid, select the X-ray visual style and rotate the design to visually inspect that all flow channels are connected.

1.34 Click Ctrl+A to highlight the design and export as an .stl file for use with the 3D-printer.

Manufacturing of base through stereolithography 3D-printing

2 Ensure the 3D-printer is clean and ready to use as per the manufacturer's instructions.

2.1 Upload a stereolithography file (.stl) to the associated 3D-printing software.

2.2 Orientate the print vertically with the ports running parallel to the Z axis.

2.3 Where required, attach support structures to the design. Note: it is important for no supports to be attached to the cover side of the design and supports must not obscure the ports.

2.4 Attach the build platform to the 3D printer.

2.5 Insert the resin tank and wiper.

2.6 Insert the resin cartridge.

2.7 The display should confirm the tank and cartridge have been inserted correctly.

2.8 The 3D printer should dispense resin into the tank and heat it to the required temperature.

2.9 Once the machine is ready, click print.

2.10 Fill a sample bag with 70% isopropanol (IMS).

2.11 When the machine has finished printing, carefully remove the 3D-printed part, and immediately place the part in the sample bag from step 2.10, ensuring that IMS flows over the part. Note: work quickly and avoid direct light exposure during steps 2.11-2.13.

2.12 Place the sample bag on a rocking platform for 10 minutes and then discard the IMS/resin mixture.

2.13 Use a pipette and eject IMS directly onto the open-faced microchannel to help clear residual resin.

2.14 Refill the sample bag with IMS and repeat step 2.11-2.13 before continuing with step 2.15.

2.15 Whilst under a fume hood, allow the IMS to evaporate fully and then expose the part in sunlight for a 24-hour period to allow for complete polymerization of the resin.

Manufacturing of laser patterned adhesive cover

3 Place the adhesive tape into the laser system bed.

3.1 Ensure the adhesive tape is lying flat and level.

3.2 The laser system should be focused to a height that is appropriate for the thickness of the material. Note: this is likely to be highly system-dependent and the user should consult the laser system manual for the appropriate recommended laser focusing parameter.

3.3 The CAD design should be uploaded to the relevant software.

3.4 The laser parameters used are frequency: 5000Hz, power: 5%, speed: 15% and 10 passes selected. Note: If an incomplete cut is observed, adjust the number of passes.

3.5 Turn on the valve to venting of any generated gases.

3.6 Click print on the software.

3.7 Click the GO button on the laser cutter.

3.8 The adhesive cover should be rectangular in shape and the edges of the cut should be smooth.

Assembly of the device

4 Polytetrafluorethylene (PFTE) tubing should be inserted into the ports of the 3D-printed base layer. Note: ensure that the tubing is ~1 mm away from the start of the flow channels.

4.1 Superglue is used to liberally cover the PFTE tubing 2 mm away from the end of the tubing, sealing it into the ports. Note: ensure the tubing is not sealed shut with glue.

4.2 Allow the superglue to fully polymerize for 24 hours.

4.3 The backing of one side of the adhesive cover is removed and the adhesive layer is placed over the base layer to seal the tubing into the ports and close the open face of the microchannel.

4.4 Run a piece of rigid material along the face of the cover to seal the device closed and remove any air bubbles.

4.5 Place the sealed device in a clamp and apply 1 tonne pressure for 3 minutes.

4.6 Allow 24 hours for the pressure sensitive tape to adhere.

4.7 Attach the tubing to the appropriate syringe pumping systems.

4.8 With a successful assembly, the injection of dH_20 at the inlet does not cause a leakage and all fluid should flow through the outlet(s).

4.9 OPTIONAL: At step 4.5, it is possible to remove the secondary backing of the adhesive cover to expose the upper adhesive side. Another IF device base layer can be stacked on top and the assembly repeated, or a substrate support such as polymethyl methacrylate can be used to increase the robustness of the device.

Characterization of the device

5 The following steps 5.1 - 5.8 are optional are provided as exemplar characterisation steps.

5.1 Vortex the neat particle suspension for 30 seconds.

5.2 Add 100 µL of neat particle suspension to 49.9 mL of phosphate-buffered saline.

5.3 Use a cell strainer with 40 µm pores and pipette the particle suspension through and count to determine the concentration.

5.4 Prime the microchannel by injecting filtered PBS at the inlet.

5.5 Inject the filtered particle suspensions at the inlet of the IF device and wait for fluid to exit the outlet tubing (this is to allow the particles to reach equilibrium focusing positions).

5.6 Collect the processed samples in microcentrifuge tubes at the outlets.

5.7 The inlet and outlets samples are quantified for particle concentration. This can be performed using a haemocytometer, flow cytometer or other cell/particle counting system (not described in this protocol).

5.8 The separation efficiency, defined as the capacity of the device to separate particles to a specific outlet and can be calculated using the following equation: [outlet sample X] / [total outlet sample] = separation efficiency (%).
